# Does sunlight drive seasonality of TB in Vietnam? A retrospective environmental ecological study of tuberculosis seasonality in Vietnam from 2010 to 2015

**DOI:** 10.1186/s12879-020-4908-0

**Published:** 2020-02-28

**Authors:** Ana Bonell, Lucie Contamin, Pham Quang Thai, Hoang Thi Thanh Thuy, H. Rogier van Doorn, Richard White, Behzad Nadjm, Marc Choisy

**Affiliations:** 1 London School of Hygiene and Tropical Medicine, WC1E 7HT, London, UK; 2 Oxford University Clinical Research Unit – Hanoi, National Hospital of Tropical Diseases, 78 Giai Phong, Hanoi, Vietnam; 3 Institute of Research for Development, 34394 Montpellier, France; 4 National Institute of Hygiene and Epidemiology, 1 Yec Xanh, Pham Dinh Ho, Hai Ba Trung, Hanoi, 100000 Vietnam; 5 National Hospital of Pediatrics, 18/879 La Thanh, Dong Da, Hanoi, Vietnam; 6 TB Modelling Group, Department of Infectious Diseases Epidemiology, London School of Hygiene and Tropical Medicine, WC1E 7HT, London, UK

**Keywords:** Tuberculosis, Seasonality, Meteorological, Sunshine, Vietnam

## Abstract

**Background:**

Tuberculosis (TB) is a major global health burden, with an estimated quarter of the world’s population being infected. The World Health Organization (WHO) launched the “End TB Strategy” in 2014 emphasising knowing the epidemic. WHO ranks Vietnam 12th in the world of high burden countries.

TB spatial and temporal patterns have been observed globally with evidence of Vitamin D playing a role in seasonality. We explored the presence of temporal and spatial clustering of TB in Vietnam and their determinants to aid public health measures.

**Methods:**

Data were collected by the National TB program of Vietnam from 2010 to 2015 and linked to the following datasets: socio-demographic characteristics; climatic variables; influenza-like-illness (ILI) incidence; geospatial data. The TB dataset was aggregated by province and quarter. Descriptive time series analyses using LOESS regression were completed per province to determine seasonality and trend. Harmonic regression was used to determine the amplitude of seasonality by province.

A mixed-effect linear model was used with province and year as random effects and all other variables as fixed effects.

**Results:**

There were 610,676 cases of TB notified between 2010 and 2015 in Vietnam. Heat maps of TB incidence per quarter per province showed substantial temporal and geospatial variation. Time series analysis demonstrated seasonality throughout the country, with peaks in spring/summer and troughs in autumn/winter. Incidence was consistently higher in the south, the three provinces with the highest incidence per 100,000 population were Tay Ninh, An Giang and Ho Chi Minh City. However, relative seasonal amplitude was more pronounced in the north.

Mixed-effect linear model confirmed that TB incidence was associated with time and latitude. Of the demographic, socio-economic and health related variables, population density, percentage of those under 15 years of age, and HIV infection prevalence per province were associated with TB incidence. Of the climate variables, absolute humidity, average temperature and sunlight were associated with TB incidence.

**Conclusion:**

Preventative public health measures should be focused in the south of Viet Nam where incidence is highest. Vitamin D is unlikely to be a strong driver of seasonality but supplementation may play a role in a package of interventions.

## Background

Tuberculosis (TB) is an infectious disease caused by the bacterium *Mycobacterium tuberculosis* (MTB), which can infect almost any organ in the body but most commonly affects the lungs [1]. Despite efforts to control TB, there were an estimated 10 million incidence disease cases in 2017 [2]. The World Health Organization (WHO) launched “The End TB Strategy” for post-2015 [3]. A key point highlighted was the need for countries to “know their epidemic” and understand the epidemiological factors playing a role in the continuing transmission of TB and therefore guide appropriate targeting of resources.

TB in Vietnam causes a high burden of disease, with an estimated annual incidence of 129 per 100,000 population [4].

Socio-economic factors, poor health system development and scarcity of medical care have been linked with increased risk of TB on individual and population levels in Asia [5, 6]. These variables alone cannot explain the seasonality of TB incidence, which has been documented in many areas of the world. Almost all studies assessing seasonality demonstrate a peak in spring/summer and a trough in autumn/winter, which is the opposite to other respiratory infections [7–11].

We are particularly interested in the role of sunlight on the variation in TB incidence as the hours of sunlight vary dramatically across the country and there is potential for interventions such as behavioral and educational interventions or vitamin D supplementation. Troughs in hours of sunlight have been linked to decreases in average Vitamin D levels and to a subsequent increase in diagnosis of latent TB [12]. A study from Wingfield et al. in Peru demonstrated a mid-winter peak in Vitamin D deficiency followed by a peak in tuberculin skin test positivity 6 weeks later and a peak in symptoms 9 weeks after that [13].

There have been few environmental epidemiological studies analyzing the seasonality of TB with respect to climate variables, but when hours of sunlight were included in the analysis, most showed a clear association [8, 13–17].

Another seasonal factor that is increasingly being explored is the interaction between infection with influenza virus and TB, where prior infection with influenza virus results in increased susceptibility to TB. This has been shown both in in vitro and clinical studies [18–20].

Although seasonality of TB has been clearly shown globally, environmental epidemiological studies to understand factors associated with this variation are limited.

Vietnam is a unique country to study the impact of climate variables on TB, as there is a high burden of disease, a large population, high levels of vitamin D deficiency and huge climatic variation across the country [21, 22].

The objectives of the study are as follows:

- determine whether there was geo-temporal variation in the incidence data for TB, by province in Vietnam;
- determine the seasonality component of incidence data for TB, by province in Vietnam;
- determine the strength of seasonality by province and identify if it varied in space;
- determine if there was an association between socio-economic, demographic, influenza-like-illness incidence or climatic variables on TB incidence.

We used mixed-effect modeling with socio-economic, demographic and climate variables to analyse the quarterly incidence of TB in Vietnam, with province and year as random effects.

By undertaking a detailed environmental epidemiological study we aim to aid future public health measures to focus efforts to reduce disease transmission and to give strength to the rationale for more clinical research in this area.

## Methods

### Study design

This is an environmental ecological study, using time series analysis to identify seasonality, harmonic regression to quantify seasonality and a mixed-effect linear model to explain the incidence of TB by province.

### Study setting

Vietnam has a large diversity in climate due to the fact that it stretches approximately 2000 km from north (22^o^N near the tropic of cancer) to south (8^o^N) and from sea level to 2000-3000 m elevation from east to west. It lies relatively close to the equator and enjoys a tropical climate. Additionally the north is exposed to four seasons, whereas the south only has two, a wet and dry season. The maximal hours of sunlight in the north peaks at the same level as the lowest rates in the south and are out of phase. In addition to this, seasonal climate factors do not have the same temporal associations north to south, that is absolute humidity and precipitation vary in different regions in a different temporal pattern than hours of sunlight. Precipitation levels are the highest in the central region.

There are 63 provinces in Vietnam and data were available for all. The median area of a province is 4600 km^2^ (IQR 2300–6800 km^2^), with a median population size of 1.175 million (IQR 0.84–1.61 million).

### Tuberculosis data

TB is a notifiable communicable disease in Vietnam. The national TB program (Viet Nam TB Information Management Electronic System, VITIMES) collects data using a passive surveillance system covering the entire country. Data is then aggregated quarterly and available by province (*n* = 63). These data were publicly available from 2010 to 2015 [23]. Classification of TB follows the WHO guidelines to include data on new cases, relapse cases, failure of treatment cases, recurrence cases and others. Any cases with both pulmonary and extra-pulmonary symptoms were classified as pulmonary TB (PTB). Cases with only extra-pulmonary symptoms are classified as extra-pulmonary TB (EPTB) [24]. Whole genome sequences from MTB were not yet available from the national program 2010–2015 and therefore those cases defined as “new cases” may be from new transmissions or from latent disease activation.

### Influenza-like-illness data

In the absence of robust data for influenza, influenza-like-illness (ILI) incidence was taken as a surrogate for influenza as a potential confounder or driver of seasonality. The global epidemiological surveillance standards for influenza, published by the WHO suggest ILI data is a useful tool in understanding trends and has been shown to correlate with influenza outbreaks [25]. ILI data from Vietnam have previously been shown to be highly seasonal [21]. These data were available from the national passive surveillance programme (29 diseases) of the General Department of Preventive Medicine (GDPM) of the Ministry of Health, monthly totals per province are publicly available [26]. These data were then aggregated by quarter.

### Demographic and socio-economic data

The General Statistics Office of Viet Nam collects and collates data on a wide variety of topics including socio-economic and health data [27]. These data have been systematically organized and made publicly available for research purposes ( [28], https://www.gso.gov.vn). The following variables were taken from this resource and were all defined per province per year: population density, proportion of male to female, proportion of urban to rural population, poverty ratio (ratio of the number of people whose income falls below ½ the median income of the country), literacy rates of 15 year olds and above, number of hospitals, immigration intake and prevalence of HIV infection at year end. Age structure was available divided into five year intervals up to the age of 80 for the year 2014. The age structure for 2014 was assumed to remain static over the 6 years of the study. We grouped the ages as young, middle and old, ≤ 15 years, 16–64, ≥ 65 years respectively, in keeping with international studies [29, 30].

### Climate data

Meteorological data was made available from the Vietnamese Institute of Meteorology, Hydrology and Environment for 67 climatic stations positioned throughout Vietnam. This includes monthly averages of daily minimum, maximum and average temperatures (°C), as well as of daily average absolute (g/L) and relative (%) humidity, cumulative precipitation (mm) and hours of sunlight. In order to compute climatic variables per province from these climatic station data, the variables where kriged [31] on a 10,000-cell regular grid (i.e. pixels of 5.75 × 5.75 km) and then aggregated by province. Kriging is a technique of spatial interpolation using a Gaussian process, whereby interpolations are weighted averages of neighboring measures. This is the standard technique in meteorology and climatology. The data from the 67 meteorological station data were used to impute the values of a 10,000-cell regular grid. Local population density was account for using weighted averages depending on the population density of each grid. The relative population densities were computed from the 2009 population density data available from WorldPop project [32]. This allowed the computation of province-aggregated climatic variables that are representative of the population of the considered province. Kriging was done by an automatic tuning of the hyperparameters of the variogram estimation as made available by the automap R package [33]. Kriging performed well overall, except for rainfall, most likely because this variable typically varies widely in space, resulting in some extreme values through kriging. These outliers were identified by comparison with the original dataset and replaced by the average rainfall for the province from the other 5 years.

Seasonal factors (meteorological and ILI data) were investigated with and without a lag of one or two quarters. This was based on findings that diagnosis of TB was made at least 12 weeks from transmission or disease activation [34]. Unfortunately, precise data on delay in diagnosis specific to Vietnam or other similar settings were not available, and therefore we assumed a similar delay in diagnosis as published. Since our data were only available quarterly we added lags so that we could examine the climate conditions at the proposed time of transmission or activation.

We followed the Reporting of studies Conducted using Observational Routinely-collected health Data (RECORD) guidelines and checklist (see supplementary material) [35].

### Analysis

All analysis, graphical representations and maps were produced using R version 3.5.0. Data from the four databases were merged by province, year, and quarter.

### Determination of geo-temporal variation in TB incidence

Incidence of TB, per province per year, was mapped using the “sf” R package [36]. Two heat maps of TB incidence, per province per quarter were produced to demonstrate and visualize seasonality in space and time (one normalized per province for temporal variation and one normalized by quarter for geographical variation).

### Determination of seasonality by province

Time series data per province were decomposed into seasonality, trend and residual components using a LOESS regression. The trends indicated in this analysis were complex and province specific and could not be modeled linearly.

For each province, harmonic regression using cosine and sine waves were used to model the seasonality on the de-trended data according to the following formula:

LOESS-detrended incidence of TB ~ a*cos (time) + b*sin (time).

### Determination of strength of seasonality by province

Where seasonal amplitude was characterized as sqrt(a^2^ + b^2^) and relative seasonal amplitude was expressed as the seasonal amplitude/mean incidence of TB ratio.

### Mixed-effect linear model: association between explanatory variables and TB incidence

A mixed-effect linear model was used, to allow both fixed and random effects to be incorporated, using the “lme4” R package [37].

All variables (except latitude, quarter and age proportions) were de-trended by province using the LOESS method described above. Year and province were introduced as random effects to control for clustering by unmeasured variables or potential trends that could remain when considering all the provinces together. All other variables were introduced as fixed effects with quarter coded as a factor and all others as continuous variables. Likelihood Ratio Tests (LRT) were used to determine significance level of each covariable. Potential confounding effects due to colinearities between covariables were controlled for by computing type-2 sums of squares (using the “car” R package [38]). Covariables were examined after detrending for normal distribution and if skewed, transformed using log transformation in order to linearize potential associations between covariables. Seasonal factors were examined with different lag times and combinations, and the final model chosen from the lowest Akaike information criteria. The full model reads:

TB incidence_*x,y*_ ~ urban_*x*_ + sex_*x*_ + population density_*x*_ + literacy_*x*_ + young_*x*_ + old_*x*_ + poverty_*x*_ + hospitals_*x*_ + HIV_*x*_ + migration_*x*_ + quarter + influenza-like-illness_*x,y-2*_ + average temperature_*x,y-2*_ + absolute humidity_*x,y-2*_ + rainfall_*x,y-2*_ + sunshine_*x,y-2*_ + latitude_*x*_ + (1|province) + (1|year).

Where *x* = province and *y* = year quarters. See Table 1 for full description of variables.

**Table 1:** All variables in final mixed-effect regression model

Variable	Definition
TB incidence	The number of new cases of TB/quarter/population of the province, in x province (x = 1,2, … 63) at y quarter (y = 1–4, in a given year), LOESS detrended
Urban	Proportion of people living in urban compared to rural areas per province per year, LOESS detrended
Sex	Proportion of male to female per province per year, LOESS detrended
Population density	Number of individuals per province divided by the geographical area of the province, per province per year, LOESS detrended
Literacy	Percentage of literate individuals in those ≥15 years of age per province per year, LOESS detrended
Young	Percentage of population under 20 years of age per province per year, LOESS detrended
Old	Percentage of population ≥ 65 years of age per province per year, LOESS detrended
Poverty	Ratio of the number of people whose income falls below ½ the median income of the country, per province per year, LOESS detrended
Hospitals	Number of hospitals per province per year, LOESS detrended
HIV	Prevalence of HIV at 31st December each year, per province, LOESS detrended
migration	Rate of in migration into a given province, per province per year, LOESS detrended
quarter	1–4, beginning in January grouping into 3 month blocks.
Influenza-Like-Illness	Number of cases of influenza-like-illness/population of the province, per province per quarter, lagged by two quarter, LOESS detrended
Average temperature	average temperature per province per quarter, lagged by two quarters, LOESS detrended
Absolute humidity	average absolute humidity per province per quarter, lagged by two quarters, LOESS detrended
Rainfall	summed rainfall per province per quarter, lagged by two quarters, LOESS detrended
Sunshine	summed hours of sunlight per province per quarter, lagged by two quarters, LOESS detrended
Latitude	Latitude value of centroid point of province
(1|province)	Province as a random effect.
(1|year)	Year as a random effect

### Model validation

The validity of the internal assumptions was assessed by examining the presence of trend, temporal and spatial auto-correlation (using the Durbin-Watson test) in the residuals.

## Results

### Determination of geo-temporal variation in TB incidence

A total of 610,676 cases of TB were reported in Vietnam from 2010 to 2015 inclusive. Of these 448,975 were classified as pulmonary TB. See Table 2 for annual incidences of pulmonary and extra-pulmonary TB as well as cases of relapse, retreatment, failure and others. The annual incidence of TB was 102.3/100,000 in 2010 and 99.1/100,000 in 2015.

**Table 2:** Numbers of cases of TB by year and category

	2010	2011	2012	2013	2014	2015	Total
PTB^a^	73,208	73,794	76,010	74,901	75,129	75,933	448,975
EPTB^a^	17,770	18,085	18,894	18,312	18,115	18,301	109,477
Relapse	6842	6925	7256	7044	7113	6546	41,726
Retreatment	382	376	494	469	468	508	2697
Failed	597	622	568	582	507	480	3356
Others	597	717	732	728	763	908	4445
Total	99,396	100,519	103,954	102,036	102,095	102,676	610,676

^a^ New diagnoses only

**Fig. 1:**
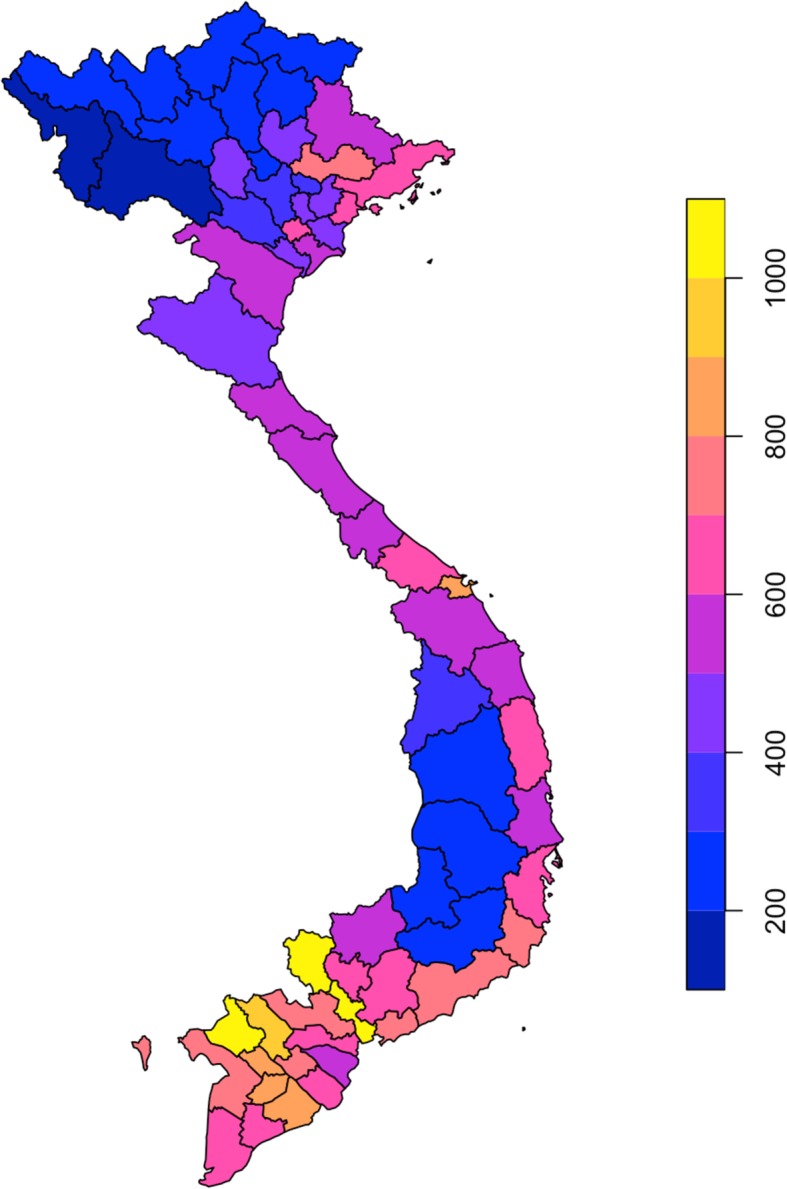
Cumulative incidence of TB per province from 2010 to 2015. Legend: Incidence per 100,000 population per province

The incidence of TB was generally higher in the southern part of Vietnam, demonstrated in Fig. 1, which shows the cumulative TB incidence by province for 2010–2015.

**Fig. 2:**
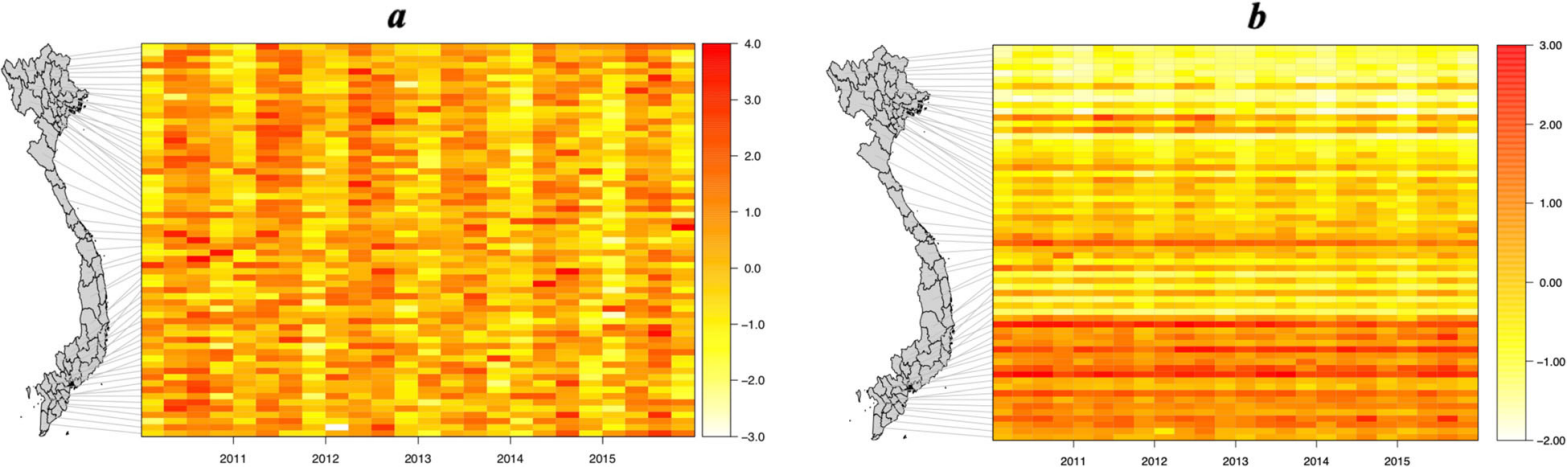
Heat maps of TB incidence per 100,000 population per province per quarter. **a** Heat map with TB incidence normalized per province to demonstrate temporal variation. **b** Heat map with TB incidence normalized per quarter to demonstrate geographical spread. Legend: Each horizontal bar on the heat map represents a province, ordered by latitude and linked to the map to demonstrate the position. Each vertical bar represents a quarter of a year (3 months) over the 6 years of the study

Heat maps of TB incidence per 100,000 population demonstrated temporal and spatial variation. Figure 2a visualizes the temporal variation and shows evidence of seasonality in all provinces over all the years. Figure 2b visualizes the geographical variation, which clearly varies with latitude.

In Fig. 2a there is a peak in the 2nd and 3rd quarters (April-Sept) and troughs in the 1st and 4th quarters (Oct-March), which is consistent with a winter trough followed by a spring/summer peak in TB incidence. In Fig. 2b the geographical variation is clearly shown.

### Determination of seasonality by province

**Fig. 3:**
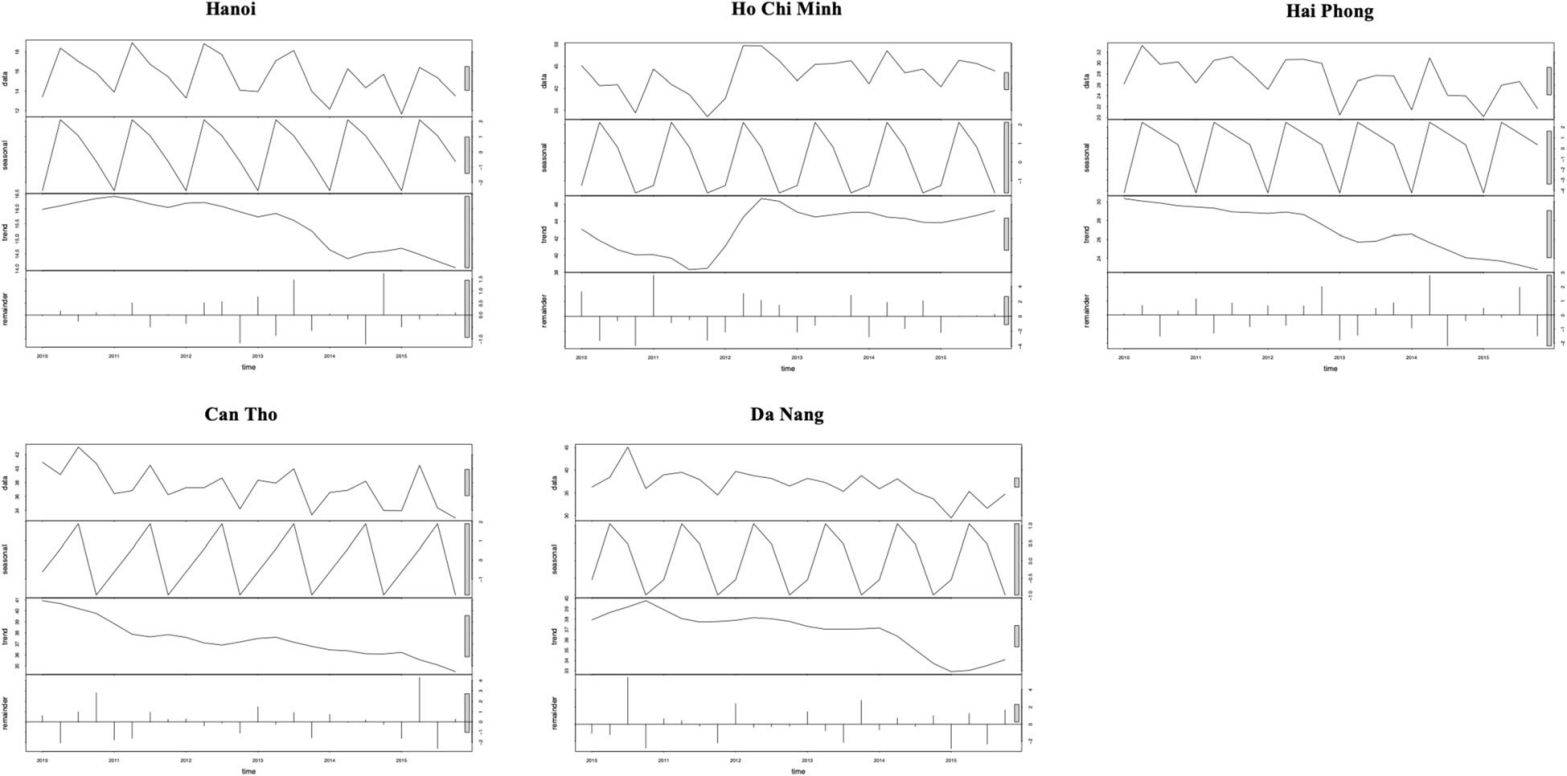
Time series plots with deconstructed components: Legend: Data Seasonality Trend Remainder

Figure 3 shows the plots of the five municipalities of Vietnam (five out of six of the largest cities). These were deconstructed using LOESS regression to give the seasonal component, linear trend and residual and demonstrate seasonality in all provinces and non-linear trends, which vary province to province.

### Determination of strength of seasonality by province

**Fig. 4:**
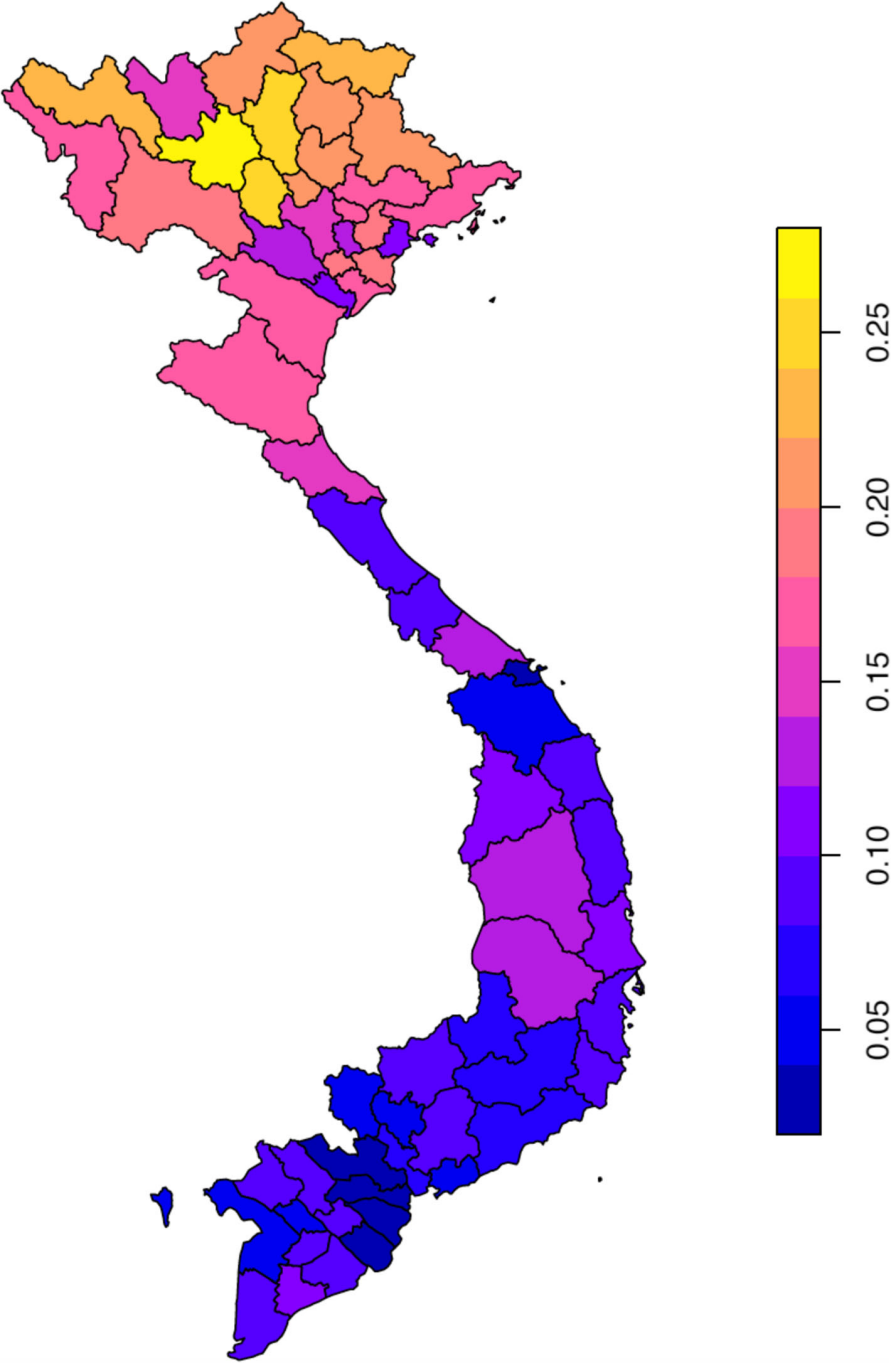
Average relative seasonal amplitude from 2010 to 2015 per province

Seasonality was then quantified using harmonic regression and the seasonal amplitude plotted in Fig. 4.

### Mixed-effect linear model: association between explanatory variables and TB incidence

As expected from the exploratory analyses, TB incidence was strongly associated with time. The second quarter, April–June was most strongly associated with increased TB incidence, increasing TB incidence by 1.7 times, 95% CI 1.08; 2.36. Interestingly latitude remained highly statistically significant with a negative correlation with TB incidence (*p* < 0.001, decreasing TB incidence by 1.0 times, 95% CI -1.39; − 0.67 with each increase in latitude). Therefore, the higher the latitude, the less TB, which is in keeping with the exploratory phase. Table 3 presents the results for the regression analysis.

**Table 3:** Results from linear mixed method regression analysis of TB incidence by province

Variable	Estimate	95% confidence intervals	Chisquare	Df	***P*** value for LRT
Urban	0.017	−0.167; 0.201	0.032	1	0.857
Sex	0.047	−1.304; 1.400	0.005	1	0.946
Population density	0.004	0.0006; 0.006	5.532	1	**0.019**
Literacy	−0.126	−0.274; 0.017	2.885	1	0.089
Young	−1.222	−1.886; − 0.561	12.406	1	**< 0.001**
Old	0.234	−0.835; 1.303	0.173	1	0.677
Poverty	0.255	−0.212; 0.724	1.135	1	0.287
Hospitals	−0.137	−0.289; 0.020	2.942	1	0.086
HIV	0.0008	0.0001; 0.002	4.898	1	**0.027**
Migration	−0.008	−0.081; 0.064	0.051	1	0.821
Quarter 2	1.721	1.082; 2.360	51.304	3	**< 0.001**
Quarter 3	0.916	0.011; 1.822			
Quarter 4	−0.231	−0.986; 0.523			
Influenza-like-illness	0.0002	−0.0009; 0.001	0.092	1	0.762
Average temperature	0.406	0.095; 0.715	6.516	1	**0.011**
Absolute Humidity	−0.482	−0.699; − 0.266	18.887	1	**< 0.001**
Rainfall	−0.00003	−0.0001; 0.00009	0.268	1	0.605
Sunshine	−0.003	−0.005; − 0.0003	4.695	1	**0.030**
Latitude	−1.027	−1.389; −0.665	29.196	1	**< 0.001**

The other co-variables associated with TB incidence were population density, percentage of those under 20 years, HIV infection prevalence, average temperature, absolute humidity and hours of sunlight. No markers of socio-economic status (literacy rates, poverty ratio, availability of hospitals) were associated with TB.

TB incidence decreased with increasing percentage of young (< 15 years) in the population (− 1.22, 95% CI -1.89; − 0.56). Hours of sunlight and absolute humidity were shown to be inversely associated with TB incidence with a lag of 2 quarters, therefore decreases in sunlight or humidity were followed 6 months later by an increase in TB incidence. Conversely, the average temperature was positively correlated at a lag of 6 months.

### Model validation

The standard deviation for the random effect of province and year were 6.0 and 0.5 respectively. Scaled residuals ranged from − 3.63 to 4.74with a median = − 0.04. Two plots were examined for any residual signal in the data, the spread of the residuals versus the fitted values and the spread of the residuals over time. Both showed even dispersal.

The Durbin-Watson test of autocorrelation gave a *p* value = 0.65, therefore we took this to demonstrate no evidence of autocorrelation.

## Discussion

TB seasonality has been observed for many years in most regions of the world where it has been studied [13–15, 20, 39–42]. Public health measures, prevention interventions and predictive models all require a robust and thorough analysis to aid understanding and accuracy. To our knowledge, this study is the first study to include socio-economic, demographic, climatic variables and ILI data to explain the seasonality of TB. This is a robust study design which adjusts for temporal and spatial autocorrelation, corrects for non-linearity and allows analysis of multiple confounders.

### Determination of geo-temporal variation in TB incidence

We found TB incidence was higher in the south than the north of Vietnam; southern border areas and large cities being the most affected. This should act as a focus for intervention efforts, potential vaccination campaigns, health education efforts, expansion of TB clinics and screening for early diagnosis.

### Determination of seasonality by province

The seasonal peaks (spring-summer) and troughs (autumn-winter) were the same across the country which was unexpected since the seasonal variation in meteorological factors do not occur in the south of the country. A study in the USA also found seasonality was present throughout all States [41]. However, of note, all States within mainland USA lie north of Vietnam, and therefore follow a more temperate climate as opposed to a tropical one.

### Determination of strength of seasonality by province

The seasonal amplitude varied by latitude with increased seasonality seen in the north. This is similar to studies from China and India [8, 43]. This implies that seasonal factors may play more of a role in the north than the south and so modifiable options, such as consideration of the role of Vitamin D may be more effective in the north compared to the south.

### Mixed-effect linear model: association between explanatory variables and TB incidence

As described elsewhere, we found a negative association between the proportion of youth and TB incidence. This could be explained in part by the difficulties in diagnosing TB in the young, where symptoms are often masked, cavitatory disease is less common and diagnostic tests are less reliable [44]. However this may also be due to an actual result, potentially explained by a sustained improvement in the Gross Domestic Product of Vietnam and the nutritional status over the last 20 years [45] in addition to established patterns of disease across the lifespan [46]. Increased population density was associated with increased incidence of TB, which could reflect increased transmission in settings where crowding occurs.

HIV infection prevalence at the province level was positively correlated with TB incidence, which is unsurprising, as the link between TB and HIV has long been established.

We found hours of sunlight, absolute humidity and average temperature were significantly associated with TB incidence at a lag of 6 months. The association between sunlight and TB incidence with a lag of 6 months, was similarly demonstrated in studies from the UK, China, and Peru [13, 15, 16]. To give perspective to the parameter estimate for hours of sunlight this was applied to the range of variation seen in Vietnam. Quarterly hours of sunlight range from 26 to 828 h, therefore if the whole population was exposed to maximum levels of sunlight there would be 2.4 per 100,000 per quarter less cases. This equates to 7.4% of cases by the 2017 annual incidence and therefore any intervention focusing on this is likely to have minimal impact.

We found an increase in average temperature was associated with an increase in TB incidence. This is similar to several other studies where temperature was explored, [8, 40, 43] including a study conducted in mainland China, but as they did not lag any climate variables, they showed an inverse relationship [14].

Our study did not find any significant association between ILIs and TB incidence. The study from South Africa, in which the seasonality of TB incidence from one hospital was analysed, demonstrated an association between peaks in influenza followed by peaks in TB incidence with a lag of 4 month [20]. This may be due to the use of ILI data and not influenza data, or the interaction may be on an individual level and not on a population level.

### Limitations & considerations

Ecological studies can be powerful studies but can fall foul of ecological fallacy. We have demonstrated an association between hours of sunlight and TB incidence at a population level, but this does not equate to an association at an individual level.

Understanding the relationship between TB incidence and climatic variables is more difficult than for other infectious diseases due to the potential time between infection and symptoms and also the added complication of disease reactivation. In addition to this complexity, the association between hours of sunlight and TB incidence does not take into account exposure. In Vietnam individuals often go to extreme lengths in order to avoid sun exposure. This is not taken into account in our model as we had no means of measuring this.

In the final model, there remained a strong association between TB incidence and latitude. This would indicate that the model did not fully explain the changes in TB incidence by the explanatory variables and there may be other important factors that need to be considered.

Data were attained from the national surveillance system, a passive system and therefore prone to under-ascertainment [47]. In addition to this, it cannot be determined whether that under-ascertainment= is consistent throughout the country and although we included a term to account for medical provision (hospitals), we cannot know if this directly translates to a similar access to care, access to diagnostic tests or level of healthcare qualification. This is likely to lead to a certain amount of selection bias, both for TB and ILI data. Since there has not been a robust assessment of the surveillance system to include proportion of missing cases, we are unable to account for this in the model.

Unfortunately the TB data were missing the age and sex of the cases of TB. The seasonality of TB has been shown to vary with age and also vary with the age structure of the province [14, 48]. Without the age of the cases we could not assess its role in confounding and or effect modification.

Another important variable that may confound the result was air pollution. Air pollution has been shown to be seasonal and to be associated with TB incidence [16]. These data were not available and due to the complexity of emissions modeling it was decided not to include any surrogate measures.

Furthermore, ILI was used as a surrogate for influenza. This is problematic as ILI may not closely correlate with influenza infections and is prone to misclassification errors. Therefore it is likely that the effect of influenza virus infection was not completely taken account of.

In view of the above, it is highly likely that there was residual confounding in the final model and this should be taken into consideration when applying the findings.

## Conclusion

TB incidence in Vietnam varied in both space and time, and preventative measures should focus on geo-temporal hotspots. Seasonality of TB was associated with reduced hours of sunlight at a lag of 6 months, but the clinical relevance of this may be minimal.

## Data Availability

The datasets generated and analysed during the current study are available at the following sites: https://github.com/choisy/ntpvn https://www.gso.gov.vn meteorological data – available on request. Programming code – available on request.

## Abbreviations

CI: Confidence interval
EPTB: Extra-pulmonary tuberculosis
GDPM: General Department of Preventive Medicine
ILI: Influenza-like-illness
IQR: Inter-Quartile Range
MTB: *Mycobacterium tuberculosis*
PTB: Pulmonary tuberculosis
TB: Tuberculosis
WHO: World Health Organization
